# Applicability of the low-grade inflammation score in predicting 90-day functional outcomes after acute ischemic stroke

**DOI:** 10.1186/s12883-023-03365-6

**Published:** 2023-09-07

**Authors:** Yang Zhou, Yufan Luo, Huazheng Liang, Ping Zhong, Danhong Wu

**Affiliations:** 1 Emergency Department, Shaoxing People’s Hospital, Shaoxing, Zhejiang Province China; 2 Department of Neurology, Shanghai Fifth People’s Hospital, Fudan University, 801 Heqing Road, Minhang District, Shanghai, 200240 China; 3 Monash Suzhou Research Institute, Suzhou Industrial Park, Suzhou, Jiangsu Province China; 4 Department of Neurology, Shanghai Yangpu District Shidong Hospital, 999 Shiguang Road, Yangpu District, Shanghai, 200438 China

**Keywords:** Low-grade inflammation score, Ischemic stroke, Inflammation, Functional outcomes

## Abstract

**Background and purpose:**

The low-grade inflammation (LGI) score, a novel indicator of chronic LGI, combines C-reactive protein (CRP), leukocyte counts, the neutrophil/lymphocyte ratio (NLR), and the platelet (PLT) count to predict outcomes of patients with various conditions, such as cardiovascular diseases, cancers, and neurodegenerative diseases. However, few studies have examined the role of the LGI score in predicting functional outcomes of patients with ischemic stroke. The present study aimed to evaluate the association between the LGI score and functional outcomes of patients with ischemic stroke.

**Methods:**

A total of 1,215 patients were screened in the present study, and 876 patients were finally included in this retrospective observational study based on the inclusion and exclusion criteria. Blood tests were conducted within 24 h of admission. Severity of ischemic stroke was assessed using the NIHSS score with severe stroke denoted by NIHSS > 5. Early neurological deterioration (END) was defined as an increment in the total NIHSS score of ≥ 2 points within 7 days after admission. Patient outcomes were assessed on day 90 after stroke onset using the modified Rankin Scale (mRS).

**Results:**

The LGI score was positively correlated with baseline and the day 7 NIHSS scores (R2 = 0.119, *p* < 0.001;R2 = 0.123, *p* < 0.001). Multivariate regression analysis showed that the LGI score was an independent predictor of stroke severity and END. In the crude model, the LGI score in the fourth quartile was associated with a higher risk of poor outcomes on day 90 compared with the LGI score in the first quartile (OR = 5.02, 95% CI: 3.09–8.14, *p* for trend < 0.001). After adjusting for potential confounders, the LGI score in the fourth quartile was independently associated with poor outcomes on day 90 (OR = 2.65, 95% CI: 1.47–4.76, *p* for trend = 0.001). Finally, the ROC curve analysis showed an AUC of 0.682 for poor outcomes on day 90 after stroke onset.

**Conclusion:**

The LGI score is strongly correlated with the severity of acute ischemic stroke and that the LGI score might be a good predictor for poor outcomes on day 90 in patients with acute ischemic stroke.

**Supplementary Information:**

The online version contains supplementary material available at 10.1186/s12883-023-03365-6.

## Introduction

Stroke is a leading cause of disability and death worldwide [1, 2]. Its incidence in China is over 336 per 100,000 people, ranking the first in the world. The mortality of stroke in China is as high as 1.7 million per year [3, 4]. Among stroke patients, approximately 80% of them are inflicted by ischemic stroke, rendering it one of the most important causes of neurological morbidity and mortality. Stroke is multifactorial in origin and influenced by multiple genetic and environmental risk factors [5–7]. Its pathogenic mechanism remains to be explored.

Systemic and local inflammatory responses play important roles in the pathogenesis of ischemic stroke [8]. An animal study has shown that humoral factors are released after neuronal death, which trigger local inflammation after ischemic injury [9]. These factors also lead to aberrant expression of receptors on microglia and astrocytes, and transgression of peripheral immune cells into the infarcted tissue [10, 11]. Furthermore, ischemic injury to vascular endothelial cells results in increased blood–brain barrier permeability and exacerbates the transgression of neutrophils, macrophages, and other leukocytes, aggravating the inflammatory response [12, 13]. Ultimately, severe neural injury is elicited and neurological deficits are manifested [14].

Previous studies have shown that age, diabetes, the baseline NHISS, and large vessel occlusion are risk factors for poor functional outcomes among ischemic stroke patients [15–17]. Recently, it has been proposed that serum biomarkers may play an important role in the prognosis of AIS in recent years [18–22]. Given the essential role of inflammation in the development of neurological deficits due to ischemic stroke, a number of inflammatory markers have been used to predict outcomes of patients with ischemic stroke. Few of them has shown high proficiency in doing so. The low-grade inflammation (LGI) score, a novel indicator of chronic LGI, combines C-reactive protein (CRP), leukocyte counts, the neutrophil/lymphocyte ratio (NLR), and the platelet (PLT) count to predict outcomes of patients with various conditions, such as cardiovascular diseases [23], cancers [24], and neurodegenerative diseases [25]. In a decent proportion of stroke patients, acute systemic inflammatory reaction to brain ischemia is superimposed on chronic, low-grade inflammation [26]. Similarly, disabled populations due to medical conditions like ischemic stroke have an increased prevalence of systemic low-grade inflammation than individuals with a greater capacity to be physically active [27].

However, few studies have examined the role of the LGI score in predicting functional outcomes of patients with ischemic stroke. Therefore, the present study aimed to evaluate the association between the LGI score and functional outcomes in patients with ischemic stroke.

## Materials and methods

### Patient selection

This is a retrospective observational study on consecutive acute ischemic stroke patients whose registry data were collected from the Department of Neurology (NR) of Shaoxing People's Hospital between January 2018 and December 2021. Inclusion criteria were: 1. age ≥ 18 years old; 2. diagnosis of ischemic stroke within 7 days after stroke onset; 3. initial onset or previous history of stroke without residual neurological deficits. Exclusion criteria were: 1. patients with severe cardiac, hepatic, or renal diseases; 2. patients with infections; 3. patients with cancers; 4. patients with immune system disorders; 5. patients who have received thrombolytic or the endovascular therapy; 6. patients with missing baseline data or follow-up data. A total of 1,215 patients were screened in the present study, and 876 patients were finally included based on the inclusion and exclusion criteria (flow chart of patient selection in Supplementary Figure 1).

Based on the rule of event per variable (EPV) for sample size estimation in logistic regression, the EPV should be 10–20 to ensure the robustness of the regression analysis results. Referring to relevant domestic studies, the preset stroke incidence was 80%, when EPV = 10, the required sample size was 15*10/0.8 = 187.5 as a total of 15 factors were included in this study. Similarly, when EPV = 20, the sample sized became 375. Given the availability of patients records and the stability of results, 876 eligible patients were included in this study.

This retrospective observational study was approved by the Ethics Committee of the Shaoxing People's Hospital (2021-K-Y-330–01) and written informed consent was obtained from all participants or their relatives.

### Data collection

At admission, baseline data, including demographic (age, gender), vascular risk factors (hypertension, diabetes, dyslipidemia, atrial fibrillation, smoking, alcohol consumption, previous stroke, and coronary heart disease), medication use (antihypertensives, antidiabetics, and statins), clinical assessment (stroke severity, blood pressure, and stroke subtype), and biochemical investigation were collected. Stroke severity was assessed at admission and one week later using the National Institutes of Health Stroke Scale (NIHSS). Systolic and diastolic blood pressure (BP) was recorded before any intervention was applied at admission. Hypertension was defined as a systolic BP ≥ 140 mmHg, diastolic BP ≥ 90 mmHg, or the administration of anti-hypertensive medications. Diabetes was defined as a fasting plasma glucose (FBG) > 7.0 mmol/L at two or more random measurements or the administration of hypoglycemic medications. Dyslipidemia was defined as the total cholesterol (TC) level > 5.18 mmol/L or triglyceride (TG) level > 1.7 mmol/L. Subtypes of ischemic stroke were categorized according to the Trial of Org 10,172 in Acute Stroke Treatment (TOAST) criteria [28]. Blood tests were conducted within 24 h of admission, including TC, TG, FBG, CRP, counts of leukocytes, neutrophil, lymphocytes, and PLT.

### Evaluation of the LGI score

The LGI score was calculated based on the following parameters: CRP, the neutrophil count, the leukocyte count, the NLR, and the PLT count. The neutrophil count was divided by the lymphocyte count to obtain the NLR. Levels of these biomarkers (CRP, leukocyte, PLT, NLR) were divided into 10 quantiles. Each score increased from 1 to 4 in the high decile (> 6th), − 4 to − 1 in the low decile (< 5th), and 0 for the middle decile (the 5th and 6th). The sum of these four scores was recorded as the LGI score, ranging from − 16 to 16 [29]. An increase in the score represented an increase in the intensity of the low-grade inflammation. For the purpose of analysis, quartiles of the LGI score were also generated using the STATA statistical software.

### Stroke related assessment

Severity of ischemic stroke was assessed using the NIHSS with severe stroke denoted by a NIHSS score > 5 [30]. Early neurological deterioration (END) was defined by an increase of ≥ 2 points in the NIHSS score within 7 days after admission [31]. Patient outcomes were assessed at baseline and 90 days after stroke onset using the modified Rankin Scale (mRS) [32]. The 90-day mRS was collected by two trained physicians through telephone interview. Based on previous studies, functional outcomes on day 90 were categorized into excellent (mRS score = 0–1) and poor (mRS score = 2–6) ones [21, 33–35]. Neurologists and physicians who participated in this study received formal training in the assessment of NIHSS and mRS. Each participant was assessed by two certified neurologists. If there was disagreement on the outcomes between these two neurologists, a third neurologist was invited to make a final decision.

### Statistical analysis

Statistical analyses were performed using the STATA statistical software (version 15.0, StataCorp, College Station, TX) and the R program (version 4.0.3, R Foundation, Vienna, Austria). Categorical variables were displayed as “number (percentage)” and compared using the chi-square test or Fisher's exact test. Continuous variables were firstly assessed using the Kolmogorov–Smirnov normality test and Levene's equal variances test. Based on the results, they were displayed as “mean ± standard deviations (SD)” for normally distributed data or “median [interquartile range (IQR)] for non-normally distributed data. Student's t-test (for two groups) and One-way ANOVA (for multiple groups) were performed for the comparison between groups that met the assumptions of normality and homogeneity of variance; Mann–Whitney U test (for two groups) and Kruskal–Wallis test (for multiple groups) were performed for the comparison of groups that did not meet the assumption of normality.

Correlation between the LGI score and the NIHSS score (baseline and one week later) was evaluated using the Spearman analysis. The efficacy of predicting clinical outcomes using the LGI score was assessed using multivariate logistic regression models to obtain an odds ratios (OR) and its 95% confidence intervals (CI). The subsequent results were adjusted by taking potential confounding factors, including age, sex, smoking, hypertension, diabetes, hyperlipidemia, atrial fibrillation, previous stroke, baseline NIHSS score, coronary heart diseases, and alcohol consumption into consideration. Sensitivity analysis was performed by stratifying participants into different subgroups, and tests of interaction were also performed by taking these factors into consideration. The area under the curve (AUC) and 95% CI were used to assess the ability of the LGI score to predict outcomes. The C-statistic, the continuous net reclassification index (NRI), and the integrated discrimination improvement (IDI) were calculated to compare the predictive ability of 2 models: the conventional model which only included the baseline NIHSS score and age, and the LGI score add-on model [25]. All reported *p* values were two-sided. *P* values < 0.05 were considered statistically significant.

## Results

### Patient characteristics

A total of 876 patients were finally included in this study according to the inclusion and exclusion criteria. The median age of the patients was 70 years [60.5,78] and 506 (58%) of them were male. The median 90-day mRS score of these 876 patients was 1. Among them, 645 patients had excellent outcomes and the other 231 had poor outcomes. Patients were further divided into four groups based on their LGI scores (Table 1). There were significant differences in their baseline NIHSS scores, baseline mRS scores, stroke subtypes, SBP, DBP, dyslipidemia, and 90-day mRS scores between groups. Compared with patients in the first quartile of the LGI score, those with increased LGI scores had higher baseline NIHSS scores (*P* < 0.001), higher SBP (*P* < 0.001), higher DBP (*P* = 0.004), higher baseline mRS scores (*P* < 0.001), higher 90-day mRS scores (*P* < 0.001), and lower ratios of small artery occlusion and other indicators (*P* = 0.002).

**Table 1:** Characteristics of ischemic stroke patients according to their quartile of LGI scores

Variables	All Patients	LGI-Q1 (≤ -5)	LGI-Q2 (-4 to 0)	LGI-Q3(1 to 5)	LGI-Q4 (> 5)	*P* value
	*n* = 876	*n* = 220	*n* = 250	*n* = 225	*n* = 181	
Male, n (%)	506 (58)	133 (60.5)	141 (56.4)	132 (58.7)	100 (55.2)	0.71
Age, years, median [IQR]	70 [60.5,78]	70 [63,76]	70 [62,78]	71 [61,78]	69 [56,78]	0.50
smoking, n (%)	256 (29.2)	59 (26.8)	79 (31.6)	59 (26.2)	59 (32.6)	0.35
drinking, n (%)	235(26.8)	63(28.6)	68(27.2)	41(18.2)	63(34.8)	0.54
Baseline NIHSS score, median [IQR]	2 [1, 4]	1[1, 3]	2[1, 3]	2[1, 4]	3[1, 7]	< 0.001***
Baseline mRS score, median [IQR]	2 [1, 3]	2[1, 3]	2[1, 3]	2[1, 3]	3[2, 4]	< 0.001***
Stroke subtypes, n (%)						0.002**
LAA	310 (35.4)	58(26.4)	92(36.8)	89(39.6)	71(39.2)	
CE	120 (13.7)	19(8.6)	37(14.8)	32(14.2)	32(17.7)	
SAA	358 (40.9)	115(52.2 )	98(39.2)	84(37.3)	61(33.7)	
Others	88 (10)	28(12.7)	23(9.2)	20(8.9)	17(9.4)	
Vascular risk factors, n (%)						
History of stroke	96 (11)	21(9.5)	25(10)	29(12.9)	21(11.6)	0.65
Hypertension	606 (69.2)	141(64.1)	176(70.4)	157(69.8)	132(72.9)	0.25
Diabetes mellitus	212 (24.2)	40(18.2)	62(24.8)	59(26.2)	51(28.2)	0.09
Dyslipidemia	385 (43.9)	83(37.7)	101(40.4)	118(52.4)	83(45.9)	0.009**
CHD	114 ( 13)	30(13.6)	33(13.2)	23(10.2)	28(15.5)	0.46
AF	93(10.6)	17(7.7)	27(10.8)	24(10.7)	25(13.8)	0.27
SBP, mmHg, median [IQR]	155[140,169]	148[134,161.5]	152[140,166]	160[144,173]	161[147,176]	< 0.001***
DBP, mmHg, median [IQR]	88[79.5,97]	87[77,94]	88[80,95]	89[82,100]	90[81,98]	0.004**
90-day mRS score, median [IQR]	1[0,2]	1[0,1]	1[0,1]	1[0,2]	1[0,3]	< 0.001***

*Abbreviations*: *LGI* Low grade inflammation, *IQR* Interquartile ranges, *NIHSS* National Institute of Health Stroke Scale, *LAA* Large-artery atherosclerosis, *CE* Cardioembolism, *SAA* Small-vessel occlusion, *CHD* Coronary heart disease, *AF* Atrial fibrillation, *SBP* Systolic blood pressure, *DBP* Diastolic blood pressure

^*^p < 0.05

^**^p < 0.01

^***^p < 0.001

### A positive correlation between the LGI score and stroke severity as well as END

**Fig. 1:**
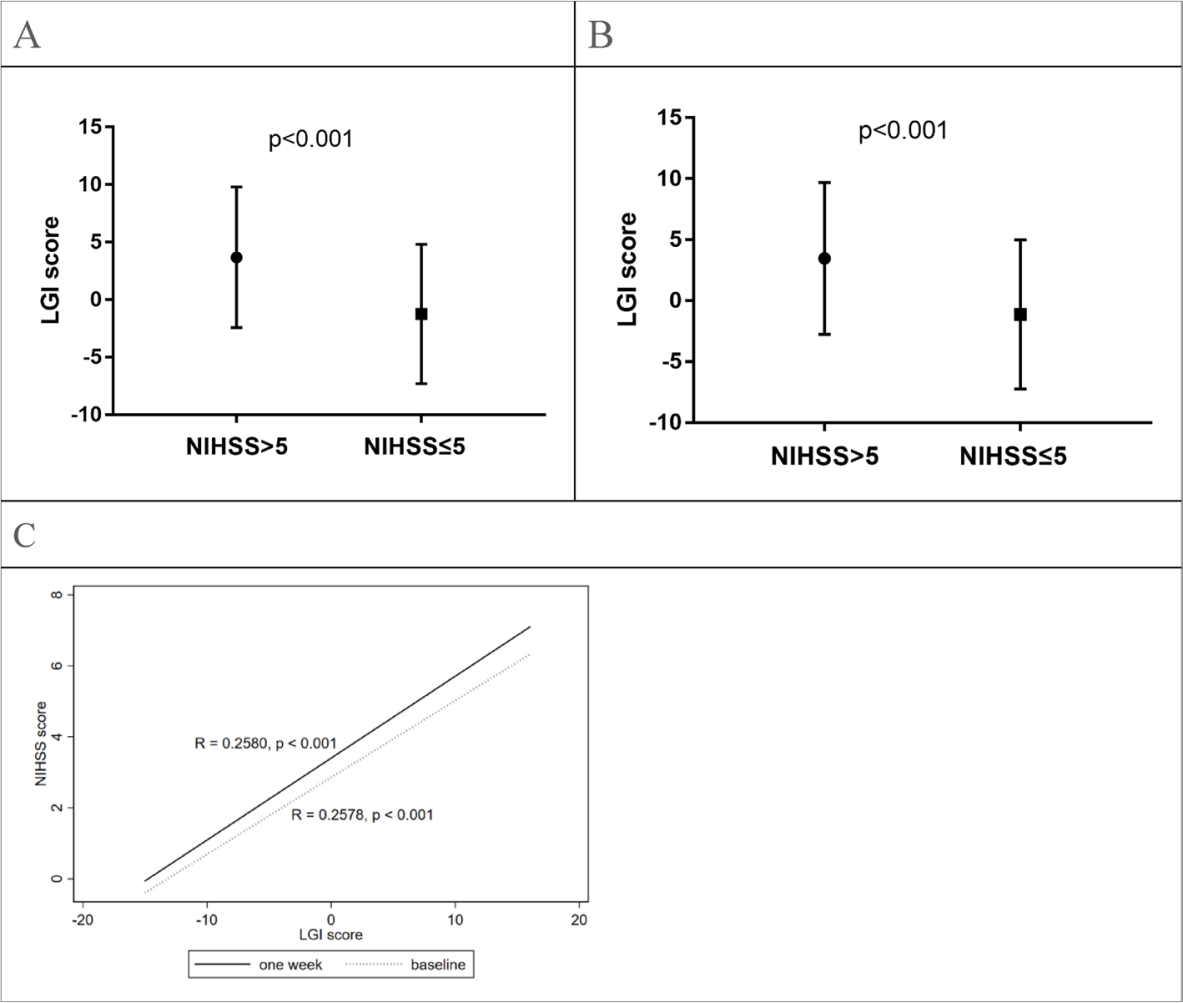
The association between the LGI score and the NIHSS score. **A** Patients with a baseline NIHSS score > 5 had a significant increase in the LGI score compared to those with an NIHSS ≤ 5 (*p* < 0.001). **B** Patients with a one-week NIHSS score > 5 had a significant increase in the LGI score compared to those with an NIHSS ≤ 5 (*p* < 0.001). **C** The LGI score was positively correlated with the baseline (*R* = 0.2580, *p* < 0.001) and one-week NIHSS scores (*R* = 0.2578, *p* < 0.001)

Based on the NIHSS scores, patients were divided into mild (NIHSS ≤ 5) and moderate/severe types (NIHSS > 5). Patients with moderate/severe stroke had a higher LGI score than those with mild stroke (Fig. 1A and B). Additionally, the LGI score was positively correlated with the baseline (*R* = 0.2580, *p* < 0.001, Fig. 1C) and the one-week NIHSS scores (*R* = 0.2578, *p* < 0.001, Fig. 1C).

Multivariate regression analysis on factors related to stroke severity and END was performed (Table 2). In the crude model, the LGI score in the fourth quartile was associated with a higher risk of severe stroke both at baseline (OR = 10.50, 95% CI: 5.55–19.86, *p* for trend < 0.001) and one week after stroke onset (OR = 7.82, 95% CI:4.32–14.16, *p* for trend < 0.001) compared with that of the first quartile. After adjusting for age and sex, the LGI score in the fourth quartile was independently associated with stroke severity at baseline (OR = 10.37, 95%CI:5.46–19.71, *p* for trend < 0.001, Model 1) and at one week after stroke onset (OR = 7.72, 95%CI:4.23, 14.10, *p* for trend < 0.001, Model 1). After adjusting for potential confounders, including demographic characters (age, sex), vascular risk factors (smoking, drinking, history of stroke, AF, CHD, HBP, DM, hyperlipidemia), the LGI score in the fourth quartile was independently associated with stroke severity at baseline (OR = 10.32, 95% CI: 5.38–19.78, *p* for trend < 0.001, Model 2) and at one week after stroke onset (OR = 7.59, 95% CI: 4.11–13.99, *p* for trend < 0.001, Model 2). In addition, the LGI score was found to be a risk factor of END (OR = 3.97, 95% CI: 1.57–10.06, *p* for trend = 0.002, Model 2).

**Table 2:** Adjusted OR for stroke severity and END in patients with ischemic stroke

	END		NIHSS score > 5 (baseline)		NIHSS score > 5 (one-week)	
	OR (95%CI)	*p* for trend	OR (95%CI)	*p* for trend	OR(95%CI)	*p* for trend
**Crude model**						
Quartile of LGI score		0.002**		< 0.001***		< 0.001***
1st quartile	reference		reference		reference	
2nd quartile	1.82(0.72,4.59)		3.05(1.59, 5.86)		1.95(1.05, 3.65)	
3rd quartile	2.38(0.94, 6.03)		4.54(2.36, 8.71)		3.42(1.85, 6.32)	
4th quartile	3.69(1.51,9.05)		10.50(5.55, 19.86)		7.82(4.32, 14.16)	
**Model 1**						
Quartile of LGI score		0.004**		< 0.001***		< 0.001***
1st quartile	reference		reference		reference	
2nd quartile	1.82(0.72, 4.59)		3.09(1.60,5.95)		1.98(1.05, 3.71)	
3rd quartile	2.43(0.96, 6.16)		4.71(2.44, 9.10)		3.57(1.92, 6.63)	
4th quartile	3.50(1.42, 8.62)		10.37(5.46, 19.71)		7.72(4.23, 14.10)	
**Model 2**						
Quartile of LGI score		0.002**		< 0.001***		< 0.001***
1st quartile	reference		reference		reference	
2nd quartile	1.98(0.77, 5.10)		2.97(1.53,5.75)		1.89(1.00, 3.58)	
3rd quartile	2.76(1.06, 7.17)		4.64(2.39,9.03)		3.48(1.85, 6.53)	
4th quartile	3.97(1.57, 10.06)		10.32(5.38,19.78)		7.59(4.11, 13.99)	

*Abbreviations*: *LGI* Low grade inflammation, *END* Early neurological deterioration, *NIHSS* National Institute of Health Stroke Scale, *OR* Odd ratio, *CI* Confidence interval

Crude model: univariate regression analyses; Model 1: gender, age; Model 2: Model 1 + smoking, drinking, history of stroke, AF, CHD, HBP, DM, dyslipidemia

^*^p < 0.05

^**^p < 0.01

^***^p < 0.001

### The association between the LGI score and poor outcomes on day 90

In the multivariate regression analysis, it was found that the LGI score was a risk factor of poor outcomes on day 90 after stroke onset (Table 3). The LGI score in the fourth quartile was associated with a higher risk of poor outcomes on day 90 compared with the LGI score in the first quartile (OR = 5.02, 95% CI: 3.09–8.14, *p* for trend < 0.001, crude model). After adjusting for age and sex, the LGI score in the fourth quartile was independently associated with poor outcomes on day 90 (OR = 5.50, 95% CI: 3.33–9.09, *p* for trend < 0.001, Model 1). After adjusting for potential confounders, including demographic characters (age, sex), vascular risk factors (smoking, drinking, history of stroke, AF, CHD, HBP, DM, hyperlipidemia), and the baseline NIHSS score, the LGI score in the fourth quartile was independently associated with poor outcomes on day 90 (OR = 2.65, 95% CI: 1.47–4.76, *p* for trend = 0.001, Model 2).

**Table 3:** Logistic regression analysis of LGI score as a predictor of functional outcomes in ischemic stroke patients

	Quartile of the LGI score (OR, 95% CI)				
	1st quartile	2nd quartile	3rd quartile	4th quartile	*p* for trend
Crude model	reference	1. 82 (1.12, 2.97)	2.57 (1.59, 4.16)	5.02 (3.09, 8.14)	< 0.001***
Model 1	reference	1.74 (1.06, 2.86)	2.54 (1.55, 4.15)	5.50 (3.33, 9.09)	< 0.001***
Model 2	reference	1.13 (0.64,1.99)	1.46 (0.84,2.55)	2.65 (1.47,4.76)	0.001**

*Abbreviations*: *LGI* Low grade inflammation, *OR* Odd ratio, *CI* Confidence interval

Model 1: gender, age; Model 2: Model 1 + smoking, drinking, history of stroke, AF, CHD, HBP, DM, dyslipidemia, NIHSS score

^*^p < 0.05

^**^p < 0.01

^***^p < 0.001

In addition, stratified analyses based on the patients’ age, gender, smoking, drinking, history of stroke, AF, CHD, HBP, DM, dyslipidemia, and baseline NIHSS scores were conducted to further examine the effect of the LGI score on functional outcomes in different populations (Table 4). However, no particular subpopulation was identified (*P* > 0.05 for all interactions).

**Table 4:** Stratified analysis of the relationship between LGI Score quartiles and the risk of poor outcomes

Subgroups	Quartile of the LGI Score (OR, 95% CI)				*p* for trend	P for interaction
	1st quartile	2nd quartile	3rd quartile	4th quartile		
NIHSS						0.969
NIHSS ≤ 5	reference	1.11 (0.64,1.93)	1.71 (0.98,2.98)	2.41 (1.34,4.32)	0.001**	
NIHSS > 5	reference	1.16 (0.32,4.23)	2.73 (0.70,10.61)	4.03 (1.08,15.07)	0.005**	
gender						0.394
male	reference	1.31(0.69,2.50)	2.19(1.17,4.11)	4.26(2.25,8.07)	< 0.001***	
female	reference	1.76(0.90,3.42)	3.64(1.83,7.24)	6.64(3.35,13.15)	< 0.001***	
age						0.797
< 65	reference	1.01 (0.40,2.52)	2.2 (0.91,5.34)	2.15 (0.83,,5.53)	0.029*	
≥ 65	reference	1.84(1.08,3.15)	2.99(1.74,5.14)	7.42(4.30,12.81)	< 0.001***	
smoking						0.413
yes	reference	0.88(0.36,2.18)	1.94(0.80,4.68)	2.94(1.20,7.21)	0.003**	
no	reference	1.89(1.10,3.23)	3.05(1.78,5.24)	6.58(3.82,11.35)	< 0.001***	
drinking						0.51
yes	reference	1.22 (0.50,2.97)	2.34 (0.94,5.80)	6.24 (2.45,15.89)	< 0.001***	
no	reference	1.65 (0.96,2.84)	2.81 (1.65,4.80)	5.08 (2.97,8.68)	< 0.001***	
History of stroke						0.338
yes	reference	4.22(0.98,18.13)	3.35 (0.78,14.46)	11.76(2.56,54.07)	0.003**	
no	reference	1.33(0.82,2.18)	2.62(1.61,4.27)	4.81(2.95,7.83)	< 0.001***	
Heart diseases						0.307
yes	reference	0.45(0.13,1.54)	2.55(0.82,7.89)	7(2.09,23.47)	< 0.001***	
no	reference	1.89(1.14,3.15)	2.86(1.71,4.78)	5.34(3.19,8.97)	< 0.001***	
AF						0.094
yes	reference	0.32(0.07,1.38)	2.71(0.71,10.36)	15.83(2.69,93.33)	< 0.001***	
no	reference	1.80(1.10,2.94)	2.62(1.59,4.32)	4.80(2.91,7.93)	< 0.001***	
hypertension						0.975
yes	reference	1.53 (0.87,2.71)	2.85(1.62,4.50)	5.44(3.10,9.54)	< 0.001***	
no	reference	1.51(0.69,3.29)	2.35(1.04,5.31)	5(2.18,11.51)	< 0.001***	
Diabetes mellitus						0.712
yes	reference	1.86 (0.70,4.96)	2.64(0.99,7.04)	5.79(2.17,15.40)	< 0.001***	
no	reference	1.41(0.83,2.38)	2.69(1.59,4.54)	5.05(2.97,8.59)	< 0.001***	
Dyslipidemia						0.381
yes	reference	1.66(0.58,4.78)	1.39(0.49,3.97)	4.79(1.67,13.70)	0.006**	
no	reference	1.49(0.89,2.49)	3.19(1.91,5.34)	5.37(3.20,9.00)	< 0.001***	

*Abbreviations*: *LGI* Low grade inflammation, *NIHSS* National Institute of Health Stroke Scale, *AF* Atrial fibrillation, *OR* Odd ratio, *CI* Confidence interval

^*^p < 0.05

^**^p < 0.01

^***^p < 0.001

### Efficacy of predicting poor outcomes on day 90 using the LGI score

**Fig. 2:**
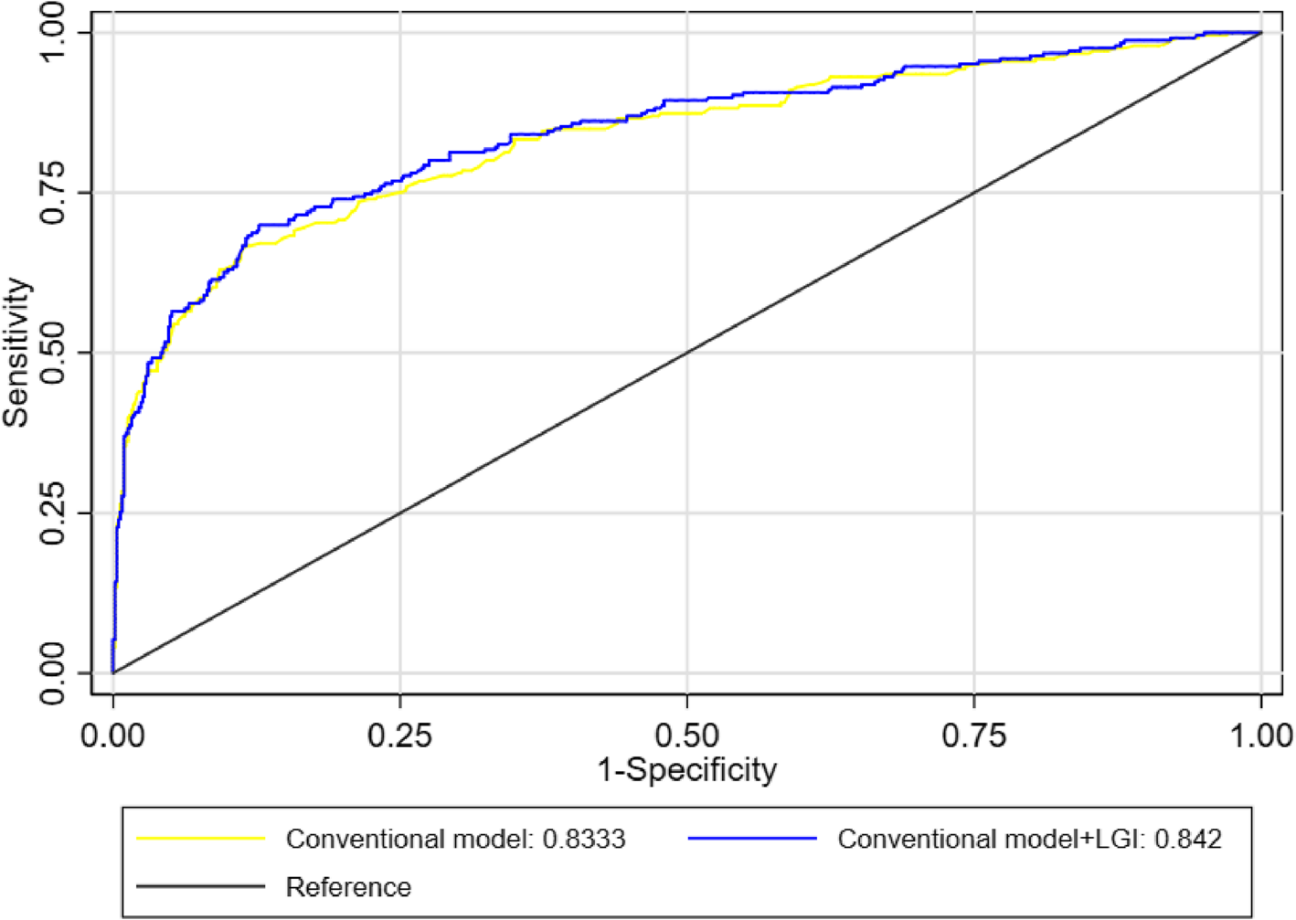
Efficacy of predicting 90-day poor outcomes using the LGI score and the conventional model

To compare the feasibility of predicting poor outcomes on day 90 using the LGI score, the ROC curve analysis was conducted. It was found that the area under the curve (AUC) for the LGI score was 0.682 (95% CI = 0.64–0.72). The area under the curve (AUC) for the conventional model was 0.833 (95% CI = 0.80–0.87) (Fig. 2), whereas the area under the curve (AUC) for the conventional model + the LGI score was 0.842 (95% CI = 0.81–0.87) (Fig. 2).

Furthermore, we assessed whether adding the LGI score to a conventional model could improve the efficacy in predicting poor outcomes on day 90. Significant improvement in the discriminatory ability of the NRI (continuous NRI = 18.7%, *p* = 0.01) and the IDI (1.34%, *p* = 0.002) were observed (Supplementary Table 1).

## Discussion

In the present study, the LGI score was positively correlated with baseline and one-week NIHSS scores. Multivariate regression analysis showed that the LGI score was an independent predictor of stroke severity and END. In addition, the LGI score was positively associated with poor outcomes on day 90 after adjusting for potential confounders, indicating that the LGI score is an independent risk factor for poor outcomes on day 90. Finally, the AUC values of LGI, CRP, WBC, NLR, and PLT for predicting poor outcomes on day 90 were 0.682, 0.6533, 0.6258, 0.6712, and 0.52, respectively, indicating that the LGI score is more superior to other single indicators in predicting outcomes of ischemic stroke patients (Supplementary Figure 2). These suggest that the currently available biomarkers of inflammation may be used together as prognostic indicators for ischemic stroke, which will facilitate our understanding of the importance of reactive inflammation in the pathogenesis of this condition. We will discuss our results in the context of leading scholars’ research findings.

It is known that inflammation is involved in the pathogenesis of ischemic stroke. Previous studies have reported that inflammation is correlated with various indicators of stroke severity, suggesting that inflammatory biomarkers may be correlated with clinical outcomes. A study reported that a low leukocyte count before treatment was independently associated with early neurological improvements [36]. Additionally, Qu et al. found that increased white blood cell counts and CRP levels after intravenous thrombolysis (IVT) were associated with poor functional outcomes in 447 patients at 3 months [37]. A cohort study and meta-analysis showed that a high NLR was positively associated with an increased risk of hemorrhagic transformation and death at 3 months after stroke [38]. Gao et al. showed that the platelet-to-neutrophil ratio (PNR) was the best predictor of poor 90-day prognosis for patients with AIS among neutrophil-related indicators [20]. Huang et al. found that an increase in the platelet-to-lymphocyte ratio (PLR) at admission was an important and independent predictor for post-stroke depression [18]. The LGI score, a more comprehensive indicator as it integrates CRP, leukocyte, neutrophil, platelet, and lymphocyte counts, potentially reflects systemic immune-inflammatory response. A previous study has shown that an elevated LGI score was associated with a higher risk of stroke recurrence independent of other vascular risk factors [39]. Results of our previous study are consistent with these findings [30, 40]. The present study further showed that the LGI score was correlated with the severity of ischemic stroke and was an independent predictor of functional outcomes at 90 days. Nevertheless, the AUC value of the LGI score was less than 0.7, indicating that these hematological biomarkers are not strong in predicting 90-day outcomes. This may be due to the fact that our study was a single-center retrospective study and included a small sample size. However, it was greater than 0.5 and higher than that of a single inflammatory indicator, suggesting that a combination of these indicators is more potent than each of these individual indicators in predicting functional outcomes of patients with ishemic stroke.

Inflammatory response plays an important role in the entire course of ischemic stroke, including initiation, progression, and recovery. In the acute phase, stagnation of blood flow distal to the thrombus may lead to release of pro-inflammatory mediators from endothelial cells of the artery, exacerbating tissue damage and disrupting the integrity of the blood–brain barrier (BBB), leading to an increase in its permeability [9]. Once these pro-inflammatory mediators enter the brain parenchyma through the disrupted BBB, neutrophils are progressively chemoattracted to the infarct zone, leading to brain injury through a variety of mechanisms, including local release of matrix metalloproteinase-9 (MMP-9), oxygen radicals, and other inflammatory mediators [41]. These factors further exacerbate damage to the BBB, increasing its permeability, and increase the risk of reperfusion injury, malignant edema, and/or hemorrhagic transformation.

Mechanisms underlying the correlation between the increased LGI score and the severity of stroke as well as functional outcomes are not clear. Activation of circulating WBC might disturb the microvascular flow and activate the PLTs that are associated with endothelial dysfunction and rupture of the vulnerable plaques [42]. Studies have shown that neutrophils are one of the first innate immune cells to respond to cerebral ischemia [5]. Activated neutrophils secrete harmful substances and inflammatory mediators that can exacerbate ischemic injury and even result in hemorrhagic transformation. Increased neutrophil counts are associated with an increased risk of new strokes, compound events, and severe ischemic stroke in patients with mild ischemic stroke or TIA [43]. In addition, other immune cells have been reported to play an important role in ischemic stroke. In a study on rats, lymphocytes have been found to coordinate the inflammatory response [44]. For example, natural killer (NK) cells were recruited to the brain by ischemic neurons and this exacerbates ischemic brain injury [45], while Treg cells appear to be beneficial by releasing anti-inflammatory cytokines, such as IL-10 [46]. As for platelets, their excessive activation and aggregation may lead to thrombosis and vascular occlusion when ischemic stroke occurs [22]. CRP, a sensitive indicator of inflammation, is closely associated with the progression and instability of atherosclerotic plaques [47, 48] which are a major contributor to the recurrence of vascular events in patients with atherosclerosis in large arteries [19]. Substantial evidence suggests a significant association between elevated levels of CRP and poor functional outcomes and mortality [49]. The LGI score encompasses these inflammatory indicators and reflects inflammation due to various causes. Given the possible synergistic effect of multiple indicators, the LGI score might be a good predictor of poor outcomes of patients with acute ischemic stroke.

Therapeutic strategies targeting the causes of inflammation of patients with ischemic stroke are attractive as they have a wider therapeutic window than the predominant approaches based on perfusion. Inhibiting neutrophils has shown to improve clinical outcomes in some experimental stroke models [50]. In a small, randomized trial including 34 acute stroke patients, Anakirna infusion over 72 h resulted in a reduction of plasma CRP and IL-6 as well as the white cell count including the neutrophil count. As a result, clinical outcomes were improved [51]. Our study proved that the LGI score is not only correlated with stroke severity and END, but also an independent prognostic indicator for poor outcomes on day 90. Adding the LGI score to the conventional prognostic model could improve the risk reclassification of functional outcomes. Furthermore, this indicator can be easily calculated from the results of routine blood tests that are mandatory upon hospital admission for every patient. To a certain extent, our findings provide evidence and guidance for clinicians in the treatment of stroke and the prediction of patients’ prognosis. The earlier patients at a high risk of stroke are identified, the earlier they will receive proper treatments and the better their functional outcomes will be. As a consequence, the burden on stroke patients, their families, and the entire society will be minimized.

To the best of our knowledge, the present study is the first to explore correlations between clinical outcomes of ischemic stroke patients and the LGI status as measured by a composite score. Indeed, this study also has a number of limitations that need to be noted when interpreting the results. Firstly, the present study is a retrospective one with participants recruited from only one hospital, which may lead to selection bias or geographically biased results. Secondly, our sample size was relatively small, and a larger sample size is needed to confirm our findings. Thirdly, we attempted to minimize the effect of confounding factors on the results, but these factors might not be completely excluded in the multiple logistic regression analysis. Fourthly, the LGI score was only measured at admission. Further studies on the impact of dynamic changes in the LGI score on prognosis of stroke patients are needed in the future. In addition, other inflammatory markers, such as tumor necrosis factor-α, interleukin-1, and interleukin-6 were not included in the LGI score due to a lack of these data.

## Conclusion

The present study demonstrates that the LGI score is strongly correlated with the severity of AIS and it is a good predictor of poor outcomes in patients with acute ischemic stroke.

### Supplementary Information

**Additional file 1: Supplementary Figure 1.** Flow chart of patient selection.

**Additional file 2: Supplementary Figure 2.** Efficacy of predicting 90-day poor outcomes using the LGI score and the inflammatory indicator(CRP, WBC, NLR, PLT).

**Additional file 3: Supplementary Table 1.** Reclassification and Discrimination Statistics for Poor Functional Outcomes by the LGI score at 90-day.

## Data Availability

The datasets used and/or analyzed to support this manuscript are available from the corresponding author on reasonable request.
