# Supplementary figures and images for: Applicability of the low-grade inflammation score in predicting 90-day functional outcomes after acute ischemic stroke
Source: BMC Neurol. 2023 Sep 7;23:320. doi: 10.1186/s12883-023-03365-6 (PMC10483771; doi:10.1186/s12883-023-03365-6)

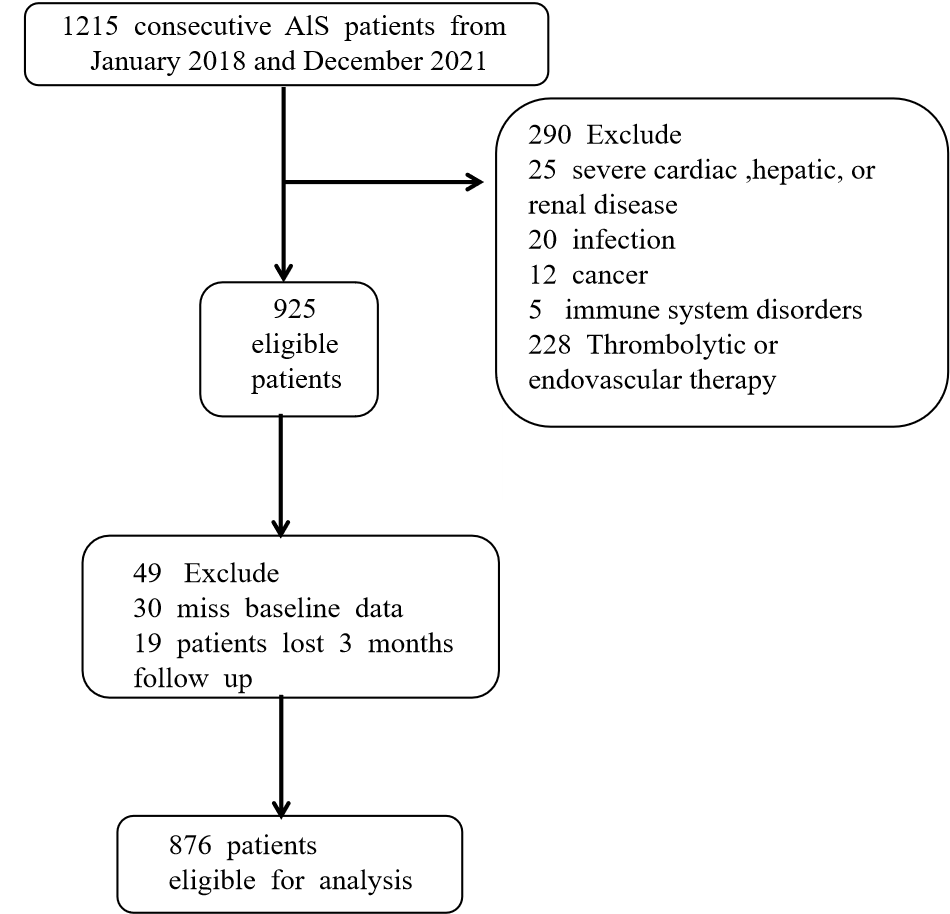


**Supplementary Figure 1. Flow chart of patient selection**

Supplement: Supplementary file 1 — Additional file 1: Supplementary Figure 1. Flow chart of patient selection. [file 12883_2023_3365_MOESM1_ESM.docx]
