# Supplementary material for: Applicability of the low-grade inflammation score in predicting 90-day functional outcomes after acute ischemic stroke
Source: BMC Neurol. 2023 Sep 7;23:320. doi: 10.1186/s12883-023-03365-6 (PMC10483771; doi:10.1186/s12883-023-03365-6)
Supplement: Supplementary file 2 — Additional file 2: Supplementary Figure 2. Efficacy of predicting 90-day poor outcomes using the LGI score and the inflammatory indicator(CRP, WBC, NLR, PLT). [file 12883_2023_3365_MOESM2_ESM.docx]

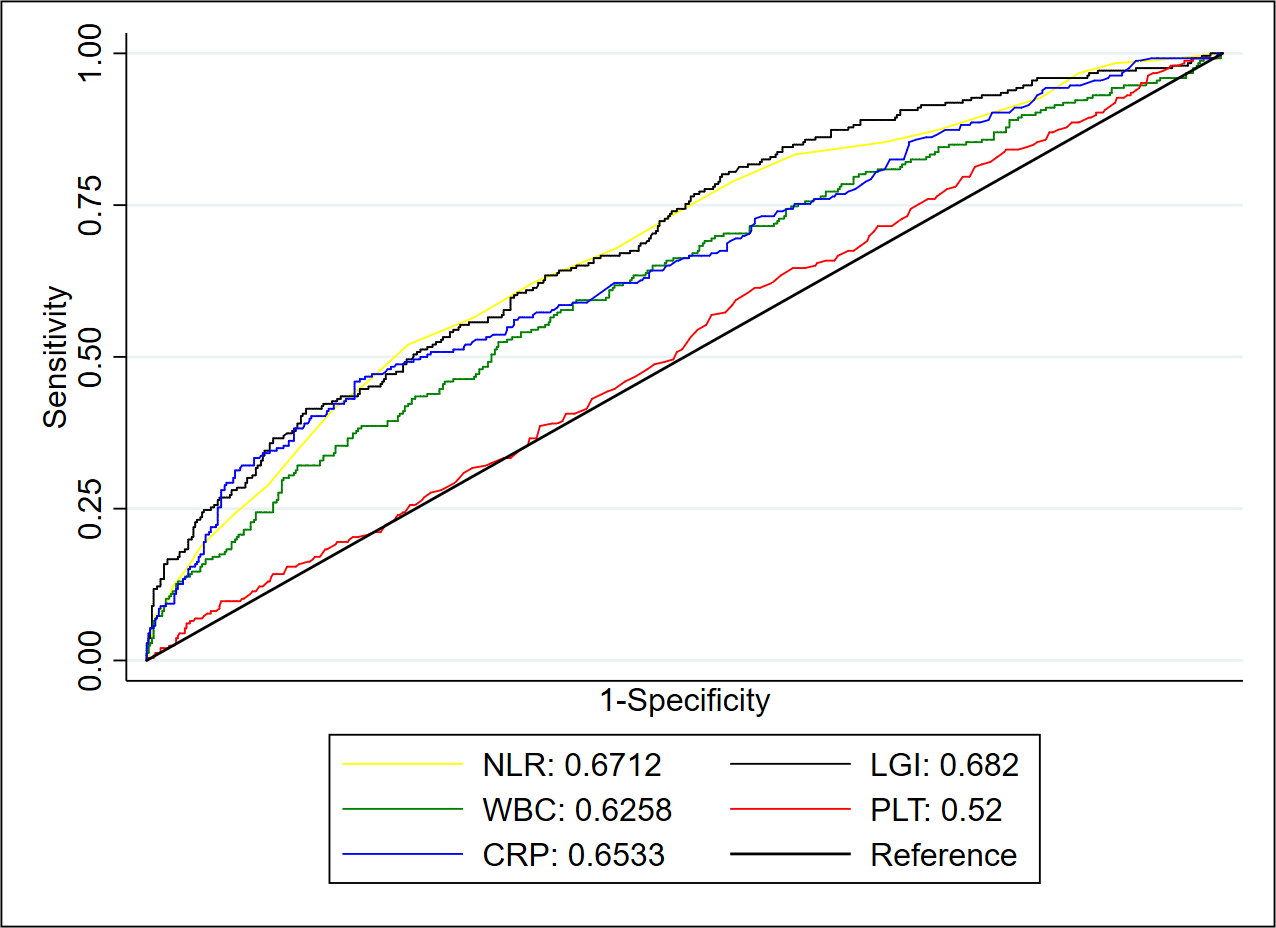


Supplementary Figure2. Efficacy of predicting 90-day poor outcomes using the LGI score and the inflammatory indicator(CRP, WBC, NLR, PLT).
