# Supplementary material for: Applicability of the low-grade inflammation score in predicting 90-day functional outcomes after acute ischemic stroke
Source: BMC Neurol. 2023 Sep 7;23:320. doi: 10.1186/s12883-023-03365-6 (PMC10483771; doi:10.1186/s12883-023-03365-6)
Supplement: Supplementary file 3 — Additional file 3: Supplementary Table 1. Reclassification and Discrimination Statistics for Poor Functional Outcomes by the LGI score at 90-day. [file 12883_2023_3365_MOESM3_ESM.docx]

| **Supplementary Table 1**. Reclassification and Discrimination Statistics for Poor Functional Outcomes by the LGI score at 90-day   \|  \| C-Statistics \|  \| Continuous NRI, % \|  \| IDI, % \|  \| \| --- \| --- \| --- \| --- \| --- \| --- \| --- \| \|  \| Estimate (95% CI) \| p-value \| Estimate (95% CI) \| p-value \| Estimate (95% CI) \| p-value \| \| Conventional model \| 0.833(0.80,0.87) \|  \| reference \|  \| reference \|  \| \| Conventional model + LGI score \| 0.842(0.81,0.87) \| 0.13 \| 18.7( 4.02 - 33.45) \| 0.01* \| 1.34(0.5 - 2.17) \| 0.002** \| |
| --- | --- | --- | --- | --- | --- | --- | --- | --- | --- | --- | --- | --- | --- | --- | --- | --- | --- | --- | --- | --- | --- | --- | --- | --- | --- | --- | --- | --- |

Note: The conventional model contained stroke severity by baseline NIHSS score and age.

Abbreviations: LGI, Low grade inflammation; NRI, Net reclassification improvement; IDI, Integrated discrimination improvement; NIHSS, National Institute of Health Stroke Scale; CI, Confidence interval. *, p < 0.05; **, p < 0.01; ***, p < 0.001.
